# User Requirements for Comanaged Digital Health and Care: Review

**DOI:** 10.2196/35337

**Published:** 2022-06-10

**Authors:** Chaloner Chute, Tara French, Sneha Raman, Jay Bradley

**Affiliations:** 1 The Digital Health & Care Innovation Centre University of Strathclyde Glasgow United Kingdom; 2 The Glasgow School of Art Forres United Kingdom

**Keywords:** delivery of health care, integrated, patient-centered care, digital technology, decision-making, health services accessibility, trust, mHealth, eHealth, telehealth

## Abstract

**Background:**

The sustainability of health and social care has led to an imperative to shift the balance of care to communities and support person-centered, integrated, preventive, comanaged, and sustainable care. The digital tool set can support this shift; however, it must extend beyond a clinical focus to include broader personal, social, and environmental needs, experiences, and outcomes. The existing digital health and care design and user requirements literature focuses mainly on specific digital products or design methods. There is little whole-system or whole-of-life consideration, which is crucial to enacting more significant transformations that span different groups and domains.

**Objective:**

This study aimed to present a set of recurring user requirements and themes for comanaged digital health and care services derived from the body of co-design projects within a digital health and care program. This study aimed to enable people and organizations looking to reorient their approach to health and care research and delivery from a system-led and condition-specific approach to a more person-centric, whole-of-life model.

**Methods:**

Participatory design formed the core methodological approach in underlying the design research, from which user requirements were derived. The process of surfacing requirements involved a selection framework for the identification of eligible projects and a structured review process to consolidate user requirements.

**Results:**

This paper presents a set of 14 common user requirements that resulted from a review of co-design projects. The findings demonstrate overlapping and reinforcing sets of needs from citizens and care professionals related to how data are comanaged to improve care and outcomes. This paper discusses the alignment, contrasts, and gaps with broader, comparable literature. It highlights consensus around requirements for personal health storytelling, sharing data on care experiences and how this can support personalized guidance, visualize trends to support decision-making, and generally improve dialog between a citizen and care professionals. These findings identify gaps around how groups and networks of people engage, posing difficult questions for people designing support services as some of the user requirements are not easily met by organizations operating in silos.

**Conclusions:**

This study proposes future recommendations for citizens as active, informed, and consenting partners using new forms of privacy-preserving digital infrastructure that puts the citizen in firm control. It is also recommended that these findings be used by people developing new digital services to ensure that they can start with knowledge of the broader user requirement context. This should inform domain-specific research and development questions and processes. Further work is needed to extend these common requirements to more explicitly consider the trust framework required when citizens comanage their data and care across a broad range of formal and informal actors. Consideration of how authority, delegation, and trust function between members of the public will be critical.

## Introduction

### Background

Health care systems worldwide face unprecedented sustainability challenges that further exacerbate the impact of the COVID-19 pandemic [1,2]. A shifting political landscape and growing recruitment crisis further affect the United Kingdom’s health care service delivery and staff well-being [3]. In parallel, there is an increasing policy and practice imperative to shift the balance of care to communities and enable a system that supports person-centered, integrated, preventive, comanaged, and sustainable care [4,5]. Scotland’s strategy recognizes digital technology as a critical asset for delivering changes at scale [6].

In previous work, the authors have proposed that the digital health and social care tool set must help systems understand people’s lived experiences [7]. This study defined individuals’ health in terms beyond what a clinical record system holds to include broader personal, social, and environmental needs, experiences, and outcomes. The authors also argued for a balance between a health care system’s need for controlled, governed, and secure record systems and a person’s need for agency, trust, choice, and the ability to connect their data across agencies, informal care circles, and communities. The authors’ *systems of record* arguments are nested inside the need for broader changes to culture and practice, from a focus on transactional relationships between citizens and systems to a more personalized and collaborative approach to care and support. In addition, the study proposed that a care system must use any formal or informal assets to sustain engagement, care interactions, and experiences on a comanaged basis. This position recognizes the complexity of the digital health and care ecosystem among the stakeholders involved, the continued exploration required to understand the efficacy of digital methods, and the challenges of various digital tools and products [7].

Therefore, this concept of the comanagement of health and social care emphasizes working in partnership with citizens to organize multiple relationships and assets to deliver person-centered care. This approach will create more sustainable methods to meet citizens’ support and self-care needs and wishes through mutual discussion and decision-making. This previous work concluded by contrasting these principles with other approaches that focused on organization-centric needs, practices, and efficiencies [7].

The literature on digital health and care design and user requirements focuses mainly on either digital products [8-16] or design methods [17-22]. This focus limits digital health and care domain knowledge to silos, such as individual clinical conditions, care groups, clinical specialties, or domains of influence (eg, health care, social care, housing, and social security). There is no whole-system or whole-of-life consideration, indicating that people looking to enact more significant transformations have no common reference to span these groups and domains. This lack of whole-system thinking makes it difficult to formulate strategies, policies, and digital architectures to satisfy the person-centered, integrated, and comanaged care ambitions set out in government health care transformation strategies described previously.

Other attempts have been made to close similar gaps through frameworks and guidance, such as the National Institute for Health and Care Excellence Evidence Standards Framework for Digital Health and Care Technologies [23] and the World Health Organization guidance on digital health for researchers [24]. However, these were limited to one system satisfying requirements related to safety and did not acknowledge the user requirements and benefits of involving citizens to create value for both the people and the system. There is a need to move beyond acceptability and feasibility to ensure that the future introduction and development of digital tools meet the identified needs.

On the basis of an analysis of insights from a co-design program, this study proposes a set of recurring user requirements and themes for comanaged digital health and care services. The findings present a starting point for further development and future research on digital health and care support options. It provides evidence of unmet needs in a whole-system context to support more holistic and integrated care led by the person.

### Related Literature

This study categorized the digital health and care user requirements literature into three main areas: (1) co-design methods to change services or elicit user requirements; (2) topic-, condition-, or product-specific design exercises; and (3) a cross-cutting review or discussion of general user requirements.

Greenhalgh et al [25] reviewed the cocreation literature, identifying different threads across disciplines, including business studies (*value cocreation*), design science (*experience‐based co‐design*), computer science (*technology co‐design*), and community development (*participatory research*). They noted commonalities across the methods that determined success. These were (1) systems thinking, (2) focus on creativity and human experience, and (3) emphasis on process, (eg, relationships, governance, leadership, and conflict). The broader literature reflects this characterization with evidence of value c-creation [17], experience-based co-design [18], technology co-design [19-21], and participatory research [22]. Additional studies reinforce the need to create end-to-end co-design frameworks that look beyond technological cocreation [26,27].

The literature demonstrates a diverse range of co-design projects covering healthy aging [8], palliative care [9], cancer care [10], medicine adherence [11], reablement [12], and a range of long-term conditions [13-16]. The precise methods vary, from those undertaking user research through semistructured interviews [8,9] to those focusing on group-based activity following user-centered design principles [10,13-15] to more product-oriented approaches [11,12,16].

The peer-reviewed literature for a cross-cutting approach to user requirements is limited, focusing on common perceptions and insights rather than user requirements [28,29]. The gray literature provides the most cross-cutting user requirements curation and analysis. This material is provided by professional membership bodies [30,31], regulators [32], or innovation and delivery agencies [33,34].

Overall, the literature focuses on design methods and co-design to elicit user requirements for specific technologies or services. However, although there are calls for more focus on whole systems, human experience, and processes, the literature does not yet fully define common user requirements across diverse groups of people, conditions, services, and technologies.

### Objective

This study aimed to address this gap by sharing a set of common user requirements based on lived experiences from a range of co-design projects across the health care continuum, which were undertaken within a digital health and care program spanning 7 years [35]. Through this, the authors sought to inform the future development of digital health and care interventions based on human experiences that span whole systems. The requirements shared in this paper have enabled the program to evolve to undertake rapid coproduction-based service modeling, prototyping and integration, and possible deployment exercises. Therefore, this study intended to support knowledge sharing to enable others to develop similar infrastructure and methods to help move beyond a purely digital product focus and satisfy the cross-cutting data-sharing, coordination, and integration needs described hereafter.

## Methods

### Overview

Before providing details regarding the specific process of identifying user requirements, it is important to provide the methodological context and the range of projects from which the requirements were derived. The reviewed projects were undertaken as part of a large-scale digital health and care program in Scotland. The Digital Health & Care Innovation Centre (DHI) was established in 2013 as a response to the need to support collaborative approaches in research and innovation across academia, civic organizations, and industry partners. The initial model of the DHI recognized the value of academic research in evidencing and testing ideas for innovation, particularly in the digital health and care context where the previous introduction of technological solutions failed to meet the needs of health care services. In addition, design-led approaches that supported rapid prototyping and testing of solutions provided the opportunity to learn quickly and iterate with the benefit of involving key stakeholders in the co-design process. During the first phase, the DHI commissioned and delivered 105 projects over 3 years. The projects reached varying stages of maturity, with some intended for concept exploration only, whereas others went into live clinical and academic trials.

Participatory design is the core methodological approach and design research practice of academics working as part of the DHI to engage a diverse range of participants in the co-design of digital health and care projects. The approach across the projects from which the user requirements were derived involved a range of methods to engage people in co-design, such as interviews, workshops, experience prototyping, creation of lived experience personas, speculative design, and the wizard of oz techniques. The methods applied within each project were bespoke to the people, topics, and outcomes in question.

### Surfacing Requirements

**Figure 1:**
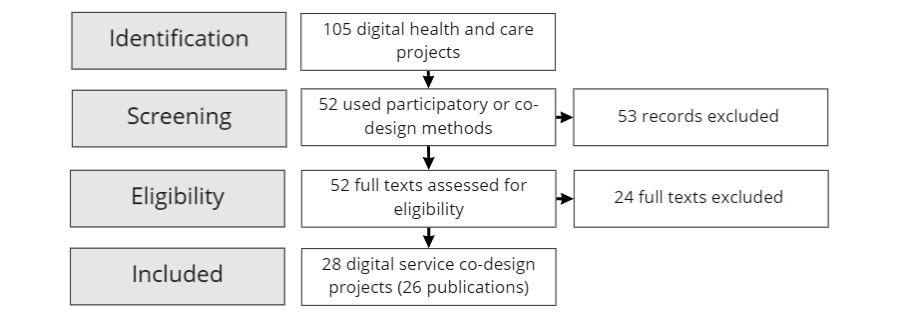
Initial study selection.

The process of surfacing user requirements, outlined in Figure 1, involved reviewing 52 co-design projects over 7 years, working with >3500 citizens, >1000 health care professionals, 16 health boards, 15 charities, and 10 social care providers. These projects supported diverse groups, generating insights across several areas, including healthy aging, mental health, and long-term condition management.

The eligibility criteria used to filter projects were as follows:

- The project focused on services where citizens were engaged in the co-design process (eg, diabetes or multiple sclerosis). Other projects were excluded if citizens were not directly engaged; for example, ambulance clinician decision support.
- The project explored communication, decision-making, and planning, which could be improved through digital tools, such as blood donation services. Other excluded projects focused on tactile, physical, and rapport-based interactions; for example, music therapy and objects to help young people communicate their needs.
- The projects were from the first 4 years of the co-design portfolio work. This criterion was introduced as the second half of the program involved co-design work that was informed by learning and experiences to date. This minimized the risk of design insights being overtly influenced by the design team because of their accrued knowledge and experiential learning.

**Figure 2:**
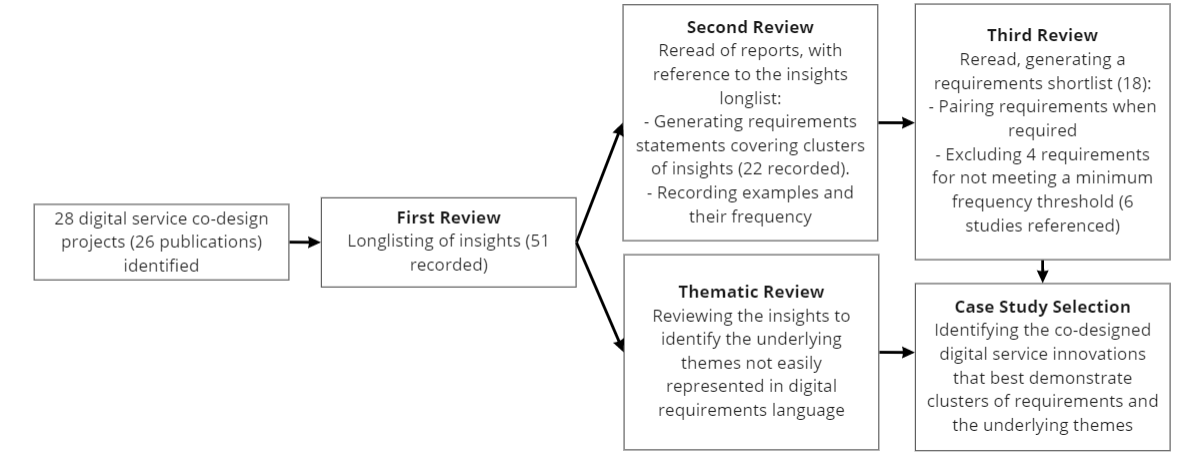
Review process.

Following project selection, the team undertook 3 reviews, as shown in Figure 2, of project reporting to gather and summarize co-design insights.

The insights were then clustered and synthesized into a set of common user requirements (>6 project references) in an appropriate user design format: “As a [type of person or role] I want to be able to [do something] to [achieve a goal].” Insights that did not fit within the user requirements language and format were analyzed thematically. Case study insights and innovative ideas from the co-design activities that showed clusters of requirements and themes in context were selected. Where possible, the outlined user requirements and themes were curated using language that does not heavily lean toward any given domain; for example, using generic language around people, personal data, dialog, and care rather than medical language around patients, clinical data, appointments, and treatment.

## Results

### Common Citizen Needs

The findings were organized into 4 parts. Tables 1-5 are a summary of the user requirements mapped to the projects that generated insights. Example quotes from the co-design participants were included to illustrate the original insights. The second part is a small section summarizing the professional needs that arose in parallel through the same co-design projects to demonstrate that in most cases, the professionals were asking for the same tools, both for the citizen and to improve activities and outcomes when providing care. The third section describes the emergent themes that could and should not be translated into the required language. The fourth section offers a case study that draws together many themes and requirements to illustrate user needs in context.

**Table 1:** Requirements: telling my story once.

Codes	Requirements (as a citizen comanaging health care services, I want to be able to)	Example quotes	Co-design studies
P1	Hold and share my personal health story and have services use this to personalize my care	“Different people every time...It can be a bit annoying I think for anyone, if you have one main doctor and you’re seeing ten other different ones, feel like you’re telling the same story over and over and over again.” [Person living with diabetes] [36] “...it would be really nice if there was a little bubble with my story there without me having to say it again and again.” [Person living with multiple sclerosis] [37]	[36-54]
P2	Share my experience and outcomes and for this to improve care for myself and others in the future	“Perhaps when I am sending notes to you, you can see, ‘yes, she cycles once a week’—or ‘she works seven days a week on her back-side!’ I think [the consultant] needs to know that people are doing some level of exercise.” [Person living with diabetes] [40]	[37, 41, 43, 48, 50, 51, 53-57]

**Table 2:** Requirements: meaningful dialog with professionals.

Codes	Requirement (as a citizen comanaging health care services, I want to be able to)	Example quotes	Co-design studies
P3	Have conversations with professionals that focus on my priorities	“It’s just trying to balance up what the patient’s needs are, versus your own agenda with them.” [Care professional] [39] “In the holistic needs assessment, the client will tick what concerns they have and will also score them out of ten. If someone’s scored something ten then that’s a really high concern for them, and that to me would be a priority” [Care professional] [48]	[38-40, 43, 48, 51, 52, 54-56, 58, 59]
P4	Have conversations with professionals who have the necessary information or test results available and gathered ahead of time	“...before I come in you would be reading [my] notes, and I’ll have a wee drop-down box with the questions I would like to ask you about my blood sugar levels, so you have [time] to think ‘oh that is what she wants to discuss today’” [Person living with diabetes] [40]	[38-43, 51, 53, 54, 56, 57]
P5	Have an ongoing dialog with professionals outside of formal appointments, allowing me to ask questions on my own terms	“...you always forget everything. The number of times I go to a clinic appointment, and I think ‘oh, I must ask them this,’ and then afterwards you go out and my mum’s like, ‘did you ask about...?’” [Person with asthma] [54]	[43,50-52,55,56]

**Table 3:** Requirements: access and understand data.

Codes	Requirements (as a citizen comanaging health care services, I want to be able to)	Example quotes	Co-design studies
P6	Access personalized guidance, signposting, and navigation support based on my personal health story	“How important is it that you can personalise the system? 100% That’s how you make it work for you.” [Older adult] [60] “That’s one of the challenges for patients, if clinical staff potentially aren’t aware of the service, it could take somebody a long time to then get engaged” [Care professional] [48]	[36, 38, 40, 42, 50-52, 54, 55, 61, 62]
P7	Have joint visualizations of clinical and personal data available to help me and others to see patterns and trends over time	“It’s all about constant monitoring and recording and using previous experience.” [Person living with diabetes] [41]	[36, 37, 39-43, 51, 53, 54, 56, 63]
P8	See a timeline or route map of my care interactions and understand their content and purpose	“I wouldn’t know who to contact or even if you phone the MS nurse, you leave a message, and they’ll get back to you but even that gets lost in translation...I do tend to write things down...I must get a book because bits of paper just go missing, I know it’s my biggest problem.” [Person living with multiple sclerosis] [38] “And also we said about having the care package—how much care is coming in and what times they are going in, because often we’d be the same—we do joint visits with carers, and you are running around trying to find out what times carers are coming in.” [Professional supporting someone living with multiple sclerosis] [38]	[38, 40, 50, 51, 55, 56, 59]

**Table 4:** Requirements: do things on my own terms.

Codes	Requirements (as a citizen comanaging health care services, I want to be able to)	Example quotes	Co-design studies
P9	Use my technology to access services and monitor myself to support my care	“Fitbits are quite trendy but [anon] is wearing something here, she’s wearing something here, she walks about with a bottle of Lucozade and sweeties so something else would drive her nuts, she just wants to fit in and be normal. A Fitbit is a good example because everybody wears one now...” [Carer of a person living with diabetes] [41]	[36, 37, 39-43, 49, 53, 54, 56, 60, 63]
P10	Manage my circle of care, communicating and sharing data with my peers, family, friends, care professionals, and community organizations	“I quite like to get advice from other mums as professionals so it’s like real-life experiences, even if those professionals have fed themselves, it’s nice to have some mums that are going through it at that particular point” [Mother] [61]	[36-39, 42, 45, 47, 50, 51, 55, 58, 60-63]
P11	Jointly manage personal, “whole-of-life” care plans with my circle of care, agreeing to actions, access rights and triggers in advance	“So there’s a team of support there but I kind of needed to hold in my head that these are all people that can be accessed. But I’m quite motivated and articulate so I have pieced together the system that works for me, and the journey has meant that different people have taken centre-stage at different times.” [Person living with multiple sclerosis] [38] “Things need to be kept local—once it goes to a big organisation they might use as evidence to say you need to go to a home...makes it more personal—a friend, a neighbour.” [Older adult] [60]	[41, 42, 45, 51-53, 55, 56, 59, 60]

**Table 5:** Requirements: use my data to unlock care.

Codes	Requirements (as a citizen comanaging health care services, I want to be able to)	Example quotes	Co-design studies
P12	Trust in how others use my personal information	“The client needs to be able to trust us to be able to get the information from them” [Care professional] [48] “...to get a hold of all of these powerful things that are in the room takes understanding and skill and compassion and it needs somebody to make it safe.” [Person living with multiple sclerosis] [38]	[36, 40, 51, 54, 58, 60, 63]
P13	Share relevant, trusted data with people who can help me	“If I need help, privacy goes out of the door” [Older adult] [58] “So to be able to have a once and for all, okay, it’s not going to be once and for all because it’s changing all the time, but a template for my story of MS with all the awful bits remembered but without having to keep on doing it with each agency you engage with, having to prove yourself.” [Person living with multiple sclerosis] [38]	[37-40,51,54,63]
P14	Have the authority to activate services that I am entitled to myself	“Although there might be things there, there was no trigger mechanism to trigger services happening” [Older adult] [62] “If it’s combined with respiratory infection, I know that so I can go to the hospital, but if it’s just cold air or air quality I’m more towards staying at home than going to the hospital.” [Person with asthma] [54] “Once (the GP) has made a diagnosis that someone has MS, that can be represented by a letter or it can be represented by a digital letter or it can be represented by a digital token and the person could carry that with them, as they do, or it could be live with (local) Council that when this person rings there’s a token that comes up to say that this is who they are.” [Person living with multiple sclerosis] [38]	[38, 47, 48, 50-54, 58, 62]

### Common Professional Needs

The co-design process often included citizens, carers, and health care professionals. Although not the focus of this paper, this section outlines the most common professional requirements and co-design projects generating these insights. This high-level summary illustrates that citizen requirements do not exist in a vacuum. In most cases, carers and professionals want the same types of data-sharing and navigation tools to help citizens and professional teams better coordinate care together (Textbox 1).

Further review is underway to explore professional comanagement needs in more detail.

**Care provider requirements.**

**Care provider requirements (as a care provider comanaging health and care services, I want to be able to)**

**Care provider requirement 1**

Access and contribute to an individual’s personal health story so that I can deliver more personalized care and enhance dialog and joint decision-making [38-43,46,48,50,53-58,63]

**Care provider requirement 2**

Share and visualize where the individual is on their current care pathway, personalized to their story to help us both manage and prepare [39,40,42,48,51,53,55,56,59]

**Care provider requirement 3**

Help me and the individual understand their condition better through the joint recording of, and access to, personal symptoms, triggers, medications, and test results [37,38,40,41,43,51,54-57]

**Care provider requirement 4**

Empower an individual with the knowledge and assets to either self-manage or escalate to other people or services [37-42,46-52,54-56,58,61]

### Additional Recurring Themes

In addition to the user requirements discussed in this paper, shared themes related to emerging principles and visions for future health care emerged during the review of the design research team’s co-design work. Although it is not within the scope of this paper to discuss these in detail, this section presents an overview of these themes to contextualize user requirements within broader transformations that are required socially, culturally, and politically to guide future innovation in health and care.

**Figure 3:**
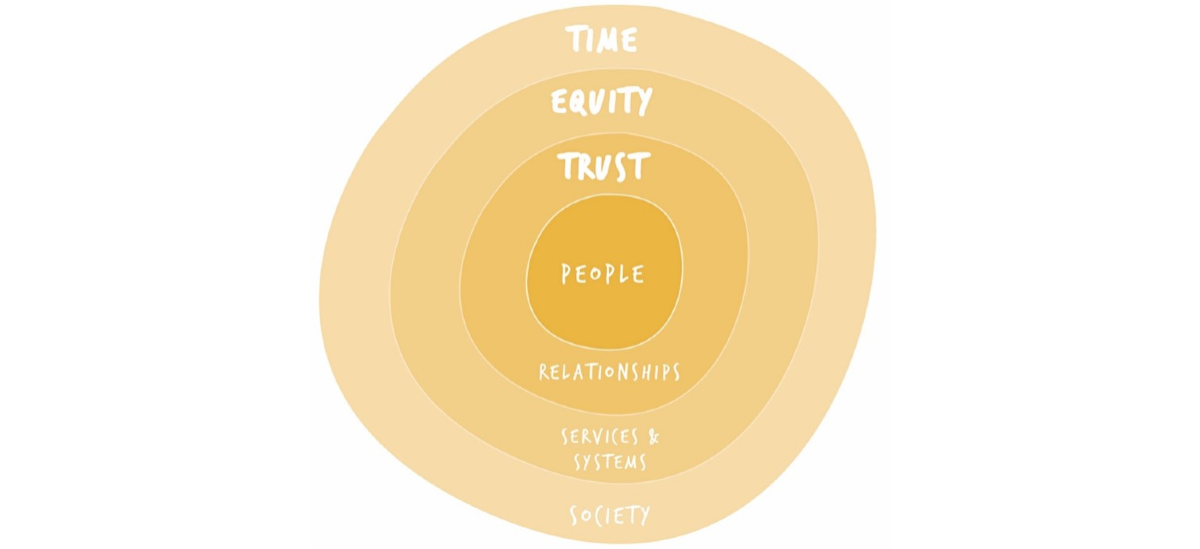
Co-design themes (image courtesy: author SR).

The emerging principles and visions for the future, as depicted in Figure 3, focused on the following:

- Enabling a person-centered focus on understanding the whole person rather than their health condition with systems built around people’s holistic needs and what matters to them
- Trust across all levels of the health care system, with a key focus on interpersonal and professional relationships
- Equity of access to information, services, and systems, revealing a tension between the need for standardization and tailoring of care
- Ensuring citizens and health care professionals have time to care for themselves and others

This paper presents an evidence base of user requirements for the future development of digital health and care interventions. However, their use will only lead to ethical and meaningful care experiences and outcomes if systemically and culturally underpinned by the values of trust, equity, and time.

### Case Study: Backpack

The *Backpack* research project provides an illustrative use case for this paper to contextualize the user requirements. The project aimed to explore how people living with multiple sclerosis would like to manage their personal information to improve their experience of accessing services and understand the potential of a person-owned data store (or digital *Backpack*) in the delivery of integrated and person-centered care [38]. The project involved engaging people living with multiple sclerosis in a focus group and a co-design workshop with health and social care professionals.

Findings from the focus group revealed the need for people to do the following:

- Retell their health stories repeatedly when accessing services and across multiple interactions with different parts of health and social care
- Understand what health and social care services are doing, including knowing about and navigating available services and keeping track of the people within their care team
- Cope with transitions and the requisite change in their care

In the first workshop, the participants designed their own physical *backpack*, which served as a relatable analogy for a person-owned record. This process included considering what information they would store and how, why, and with whom they would share it. In the final workshop, health and social care professionals set typical health care tasks to explore how person-owned records might change the way they work through paper-based and digital prototypes. Through these activities, 4 concepts emerged regarding the future use of person-owned records. Table 6 outlines these concepts and relates them to the defined common user requirements.

The findings from the Backpack project are evident in many of the requirements shared in this study, as illustrated in Table 6.

**Table 6:** Backpack innovation concepts.

Co-designed innovation concept		Related user requirements
Concept	Description	
Circle of care	This is a digital means of mapping interactions with formal and informal health and care systems. The complex, multi-organization services accessed by people living with multiple sclerosis (MS) means that it can be challenging to understand who they are seeing or have seen and for what purpose. The same issue occurs for the health and social care professionals who provide care, leading to unneeded or duplicated referrals, poor resource use for the health care system, and unhelpful interactions for the citizen. Participants discussed “building your own care team”—mapping interactions with the person at the center of a connected network of professionals and a timeline showing who they saw or will see. By mapping what has happened and what will happen, the “backpack” should create a space for shared and shared decision-making, leading to a more equitable relationship between care providers and people who access support. Circle of care technologies are beginning to emerge in practice, for example, to support care for parents and children [64].	P8, P10, P11, and CP2
Health story	Citizens can tell their own health stories using their choice of format and content. Participants often found that professionals lacked even basic knowledge about them but did not wish to recount their stories repeatedly. Professionals saw value in understanding the citizen and their needs before meeting for the first time. A health story might be text or video, contain key dates (eg, diagnosis or change in personal circumstance), and can be updated. It may also contain other suitable information, such as a video of their home environment. A health story would be shared by consent from specific organizations or publicly (which might help others in similar situations). These preshared health stories have been shown to improve communication between care professionals and the people they are supporting [65].	P1, P3, and CP1
Automatic form filling	This is a digital means of avoiding repeated form filling. Existing data in a person’s backpack could autofill many form fields. In particular, the data could be used to identify eligibility criteria quickly and easily. This method would replace the need to “make yourself known” to various health care providers to find out what services are available to an individual. This mechanism would gradually fill up, collecting data as it went, and would not require a massive data entry exercise at the beginning. The stored data could be used, with the person’s consent, to find eligible services automatically. Participants also suggested an “in case of emergency” feature to share their backpacks with nominated people if necessary.	P6, P13, and P14
Responsive case management	This includes digital tools that help professionals in sharing information and caring for people with person-owned records. A regional MS nurse does not necessarily know about any change in circumstance for the people they support. They may not be informed of hospital admissions, deterioration in a condition, or even death. This mechanism was visualized as a list of all people with MS in the region. The MS nurse can send messages to individuals or a subset that matches chosen criteria. The MS nurse could order the patients according to criteria and be alerted to any change of circumstances entered in the “backpack” by citizens. This mechanism would allow the MS nurse to effectively help many people with MS. Comanaged digital tools are now being studied, which use wearable data and patient-reported outcome measures to help clinical teams identify and respond to change [66].	P2, P5, P6, CP1, and CP3

## Discussion

### Principal Findings

This study provides a robust and systematic analysis of common user requirements for the digital comanagement of care. On the basis of a diverse body of co-design work, it provides a starting point for people and organizations looking to reorient their approach to health care data sharing from an organization-centric to a person-centric model. This study set out to create an initial frame of reference for *whole-system* service and system design, underpinned by insights generated through co-design with a wide range of user groups across multiple domains. These findings demonstrated a consistent set of user requirements that look beyond individual technologies and processes specific to one type or domain of care. Through the active participation of both citizens and care professionals in the underlying design research, the findings also demonstrated overlapping and mutually reinforcing sets of needs from both groups related to how data are comanaged to improve care and outcomes.

### Comparison With Prior Work

The peer-reviewed literature focuses mainly on co-design methods and technologies for individual health and care services. The work comparable with this synthesis was extremely limited, with some studies focusing on common perceptions and insights [28]. Other studies elicited more definitive requirements for more complex needs but still with an individual product focus [24]. More systematic user requirements curation and analysis were primarily found in the gray literature provided by professional membership bodies, regulators, or innovation and delivery agencies. As a result, although these pieces were broader in scope, they were still tied to individual domains, mainly medical [30,33] and social care record keeping [32]. Table 7 maps these 5 comparators against the common requirements described in this study. It identifies the areas of complete alignment and partial alignment.

In contrast to the approach shared in this paper, which focused on co-design insights generated separately from any given product, Vo et al [28] analyzed 43 studies reviewing mobile health (mHealth) apps providing commentary on existing apps. However, the analysis rose above individual products or methods, focusing on the common strengths and weaknesses of mHealth apps. This study’s concepts map well relative to our findings, particularly regarding personalization, meaningful dialog, and citizen participation. Without digital tools that rebalance the power dynamic between citizens and professionals, citizens may otherwise “resign themselves to receiving care without taking up the possibility to engage in active participation” [28]. Both their work and ours found that citizens wish to use these digital tools to facilitate relationships and not to replace them. Finally, the study identified concerns about the scientific validity of some mHealth apps, which were not covered within our user requirements’ elicitation [28].

The cross-cutting needs for patients identified by Bhattacharyya et al [29] arrived at a broadly comparable set of user requirements. The key features were again mapped to the most common requirements in this study, focusing on elements relating to trend analysis, navigation, and guidance. All key features were covered by the common requirements presented in this study [29].

Further overlap was evident in the set of benefits and other supporting materials of interviews and focus groups on the topic of personal health records. However, the findings focused on transactional National Health Service (NHS) service access (beyond the scope of this work), such as reminders for medications or access to test results [30].

In the context of previous work on user needs relating to personal health records, there was further alignment with the user requirements, particularly the data-sharing relationship between a patient and clinical team, but with an additional focus on trust and privacy not readily evident in the broader user requirements literature. Another parallel was the account of complementary professional needs in the comanagement of data. A wider NHS work identified additional requirements beyond the findings in this paper around the delegation of authority over data and ethical limitations to medical data sharing [31,33]. Equivalent exercises in the social care domain focused on general shared care record methods, prioritizing more joint care team capabilities to improve citizen outcomes [32].

Overall, the requirements aligned with previous research and strongly in the case of requirements for personal health storytelling, sharing data on health experiences and how this can support personalized guidance, visualizing trends to support decision-making, and generally improving dialog between a citizen and a care professional (a *vertical* relationship). However, there were notable differences where this study makes key contributions. The first contribution was the new knowledge curated, with common requirements identified in this paper, extending to cover more *horizontal* relationships and more holistic needs beyond dialog with any one professional; for example, the need to create care plans and manage care circles involving multiple professionals, informal carers, agencies, and technologies. Another example was the ability to comanage the data itself, with personally held data being trusted by professionals and, in turn, professionals being trusted by citizens to use their personal data appropriately.

There were some contrasts and gaps; for example, only this study identified that citizens needed to have authority granted to them and the data they hold. This authority was crucial to creating a more effortless experience in demonstrating eligibility when moving between professional domains (eg, for a benefit or being able to access rationed specialist services directly). Overall, the differences were related to the scope of the different pieces of work. The review of projects was concerned with the whole of life and integrated services and, thus, reflected requirements that spanned domains. Previous studies, acting out of only one domain, tended to reflect requirements that optimized citizen-professional dialog within that domain, service, or specialty [30-33].

The second key contribution was related to this method. This study and the summarized evidence covered 3 main elements that were not entirely paralleled by any of the previous key work comparators. For example, this paper has reviewed a large body of co-design projects, considered citizen and professional needs in tandem, and generated specific user requirements through this review (Table 8).

This finding points to the need for robust literature that summarizes and translates large bodies of co-design input into requirements language to support the comanagement of care at the whole-system level.

**Table 7:** Common requirements comparisons across publications.

Common requirement (authors)	Vo et al [28]	Bhattacharyya et al [29]	Royal College of Physicians [30]	NHS^a^ Digital [33]	Care Quality Commission [32]
Hold and share my personal health story and have services use this to personalize my care	Partial alignment	Complete alignment	Complete alignment	Complete alignment	Partial alignment
Share my experience and outcomes—and for this to improve care for myself and others in the future	Complete alignment	Complete alignment	Complete alignment	Complete alignment	Complete alignment
Have conversations with professionals that focus on my priorities	Complete alignment	Partial alignment	Complete alignment	N/A^b^	Complete alignment
Have conversations with professionals who have the necessary information or test results available and gathered ahead of time	Partial alignment	N/A	Complete alignment	Complete alignment	Complete alignment
Have an ongoing dialog with professionals outside of formal appointments, allowing me to ask questions on my own terms	Complete alignment	N/A	Complete alignment	Complete alignment	N/A
Access personalized guidance, signposting, and navigation support based on my personal health story	Complete alignment	Complete alignment	Complete alignment	Complete alignment	Complete alignment
Have joint visualizations of clinical and personal data available to help me and others to see patterns and trends over time	N/A	Complete alignment	Complete alignment	Complete alignment	Complete alignment
See a timeline or route map of my care interactions and understand their content and purpose	N/A	Complete alignment	N/A	Partial alignment	N/A
Use my technology to access services and monitor myself to support my care	Complete alignment	N/A	Complete alignment	N/A	N/A
Manage my circle of care and communicate and share data with my peers, family, friends, care professionals, and community organizations	N/A	N/A	Partial alignment	N/A	N/A
Jointly manage personal, “whole-of-life” care plans with my circle of care, agreeing to actions, access rights and triggers in advance	N/A	Complete alignment	N/A	Partial alignment	N/A
Trust in how others use my personal information	Complete alignment	N/A	Partial alignment	Complete alignment	Complete alignment
Share relevant, trusted data with people who can help me	N/A	N/A	Complete alignment	Complete alignment	Complete alignment
Have the authority to activate services that I am entitled to myself	Partial alignment	N/A	N/A	N/A	N/A

^a^NHS: National Health Service.

^b^N/A: not available.

**Table 8:** Comparison of study elements.

Study element	Chute et al [7]	Vo et al [28]	Bhattacharyya et al [29]	Royal College of Physicians [30]	NHS^a^ Digital [33]	Care Quality Commission [32]
Reviewed large body of design studies	Yes	Yes	No	No	No	Yes
Considered citizens and professionals in tandem	Yes	No	No	No	Yes	Yes
Generated specific user requirements	Yes	No	Yes	Yes	Yes	No

^a^NHS: National Health Service.

### Implications for Practice

The requirements summarized in this paper pose difficult questions for people designing health care, social care, and broader support services as they are not easily met by organizations operating in silos. For example, the most universal of all the studied citizens’ needs was that of people wanting to *tell their story once* and not repeat themselves across different parts of the system. Although there were numerous initiatives to create a joint approach, they rarely looked across domain boundaries. This problem is best illustrated by the ongoing pursuit of a single clinical record and the domain-specific goal of aggregating all clinical data to drive improved care and outcomes. This single medical record would undoubtedly help health services and individual patients have more joint medical care. However, it would not meaningfully change the way citizens transact with social security and housing or empower their informal circle of care or a third or independent sector organization to support their nonmedical needs. A record dictated by a medical model and associated standards and governance is not likely to tolerate new forms of data generated by citizens, broader organizations, and other sources that would increasingly allow for more context-rich, whole-of-life outcomes to be pursued through greater personalization and prevention.

However, a citizen-comanaged, holistic story would be heavily dependent on the quality of the organizational systems and the data it must synchronize with. A prerequisite for more citizen control and reuse of their medical records requires that those records be well-defined and structured and that those supplying health care software conform to modern, standards-based practices. In technical terms, these challenges are beginning to be met at scale by the proliferation of application programming interfaces, messaging standards (eg, Fast Healthcare Interoperability Resources [FHIR]), and data storage models (eg, OpenEHR). The technical barriers are increasingly surmountable; however, a more significant effort will be required in evolving the culture, commissioning, and supplier practices to adhere to standards and separate the data from software products to enable its reuse.

The routes available to meet the user requirements outlined in this paper will almost certainly do so with the citizen as an active, informed, and consenting partner using new forms of privacy-preserving digital infrastructure that puts the citizen in firm control. Only through this kind of comanagement of data can comanagement of care that respects *whole-of-life* needs and satisfies whole-system governance and trust be achieved. The findings in this study can be used by people developing new digital health and care services to ensure that they can start with knowledge of the broader user requirement context. This should inform domain-specific research and development questions and processes. For example, when creating a shared care record between health and social care, the citizens’ requirements in this study may encourage system designers to consider how the record needs to be viable beyond either of those 2 domains. This additional consideration may help us collectively move toward systems that support citizens to *tell their story once* and reuse the record to access broader support services.

### Limitations and Future Work

There are three main limitations to this study and several ways the authors attempted to mitigate them.

First, all source projects were undertaken by the same design research team from one institution (with other universities occasionally collaborating). Therefore, although diverse, the methods and results came from the same collective approach to co-design, which may have limited the general applicability of the outputs. A comparative review of the broader literature, as documented in the *Introduction* section, aimed to test the findings in a broader context to mitigate this.

Second, as a design research group, knowledge and design experience grew. Therefore, later work was often informed by earlier work, which may have created patterns based on the interventions. This study was limited to reviewing the first 4 years of work to mitigate this effect. Further ≥30 recent projects have not been included to avoid more recent work artificially inflating the perceived commonality of these requirements.

Finally, most of the design research was commissioned by the NHS. Although many participants were in the social care and third sector, the overall tone of most of the work was health (clinical care) focused. The team strove to take a step back from the initial commissions and used design methods to ensure that the project asked the right questions across diverse populations in a broader context of health, care, and well-being. However, the overall tone is undeniably still clinical care focused; thus, future work is needed to expand the understanding of nonclinical common user requirements to complement the findings of this paper.

Future research on co-design and requirements elicitation could build on this foundation and address several gaps. For example, although *circle of care* and *joint care planning* were common requirements, they are both concepts that span many people and organizations. Therefore, more work is required to harmonize requirements and data sets across multiple actors. It is also unclear where peer networks (eg, diabetes management community networks) end and circles of care (eg, friends, family, and carers) begin and the level of data sharing and privacy that relates to these different types of relationships. Finally, the concept of delegation of authority has begun to emerge as health care systems become more digitally enabled. To support equity of access and maintain interpersonal care relationships, some groups will need to name and delegate authority to trusted people, who can then act on their behalf with digital services.

### Conclusions

This paper demonstrated common user requirements relating to the comanagement of care between citizens and their circles of care. The common requirements relating to *vertical* relationships between a citizen and a professional were corroborated by comparator literature. The common requirements extended to cover the *horizontal* relationships between people and their broader support networks across services and agencies and their informal circles of care. Further work is needed to extend these common requirements to more explicitly consider the trust framework required when citizens comanage their data and care across a broad range of actors. Consideration of how authority, delegation, and trust function among members of the public will be critical. The authors propose that these user requirements can inform service design and data-sharing infrastructure across organizations involved in providing health, social care, and well-being support. We welcome further dialog on how these requirements can drive a person-centered integration agenda that brings value to people and the system.

## Abbreviations

DHI: Digital Health & Care Innovation Centre
FHIR: Fast Healthcare Interoperability Resources
mHealth: mobile health
NHS: National Health Service
